# The wild ways of conscious will: what we do, how we do it, and why it has meaning

**DOI:** 10.3389/fpsyg.2013.00574

**Published:** 2013-09-03

**Authors:** J. Scott Jordan

**Affiliations:** Director, Institute for Prospective Cognition, Department of Psychology, Institute for Prospective Cognition, Illinois State UniversityNormal, IL, USA

**Keywords:** entrainment, teacher/student interaction, mimicry, imitation, synchrony, mirroring, mirror neuron system

## Abstract

It is becoming increasingly mainstream to claim that conscious will is an illusion. This assertion is based on a host of findings that indicate conscious will does not share an efficient-cause relationship with actions. As an alternative, the present paper will propose that conscious will is not about causing actions, but rather, about constraining action systems toward producing outcomes. In addition, it will be proposed that we generate and sustain multiple outcomes simultaneously because the multi-scale dynamics by which we do so are, themselves, self-sustaining. Finally, it will be proposed that self-sustaining dynamics entail meaning (i.e., conscious content) because they naturally and necessarily constitute embodiments of context.

While the present paper addresses the relationship between consciousness and action control, its ultimate goal is to propose that terms such as “action” and “consciousness” are scientifically inadequate and, in the end, may have to be replaced in a scientific account of what we do, how we do it, and why it has meaning. This is because, as I will argue, the current conceptual framework used in cognitive science (e.g., perception, cognition, action, attention, intention, and consciousness) is not capable of addressing the complex array of causal regularities that have been discovered in cognitive science over the past 30 years.

In addition, the current conceptual framework has yet to give rise to a scientific conception of how we do what we do that renders the phenomenon of “consciousness” a *necessary* aspect of the causal story. That is, consciousness is described as either identical with the physical (i.e., identity theory), emergent from the physical (i.e., emergentism), as an informational property of causal relations (i.e., functionalism), or as an aspect of reality other than the physical (i.e., double-aspect theory and property dualism). In all of these positions, consciousness is not a logically necessary aspect of the causal story. That is, the scientific, causal description of how we do what we do is able to disregard consciousness as a causal factor.

While the notion that consciousness might not be logically necessary is certainly popular, one might also take it to indicate the need for an approach to “how we do what we do” that renders consciousness causal (i.e., non-ephiphenomenal). In what follows, I present Wild Systems Theory as an approach to causality and consciousness that renders the latter logically necessary. To be sure, by the time this has been explicated, the term “consciousness” will mean something different that what is referred to via constructs such as Access Consciousness, Metacognition, and Phenomenal Consciousness (Block, 1995, 2001; Cleeremans, 2005).

## What we do

One of the reasons consciousness is not seen as logically necessary in scientific accounts of “what we do” is because we do not conceptualize it as an activity. In contemporary cognitive science, “what we do,” is conceptualized via terms such as perceive, act, think, attend, intend, infer, cognize, represent, remember, simulate, and behave. Notice that all of these terms are verbs. When the concept “consciousness” is thrown into the mix, it enters as a noun. In short, consciousness is not conceptualized as something we do.

In the early days of experimental psychology, this was not the case. In fact, consciousness was seen as an *act* of intending, “… all experience involves directedness toward an object… Every mental phenomenon includes something as object within itself” (Ash, 1995, p. 28), and there was much theory regarding “conscious acts,” not in the sense that consciousness caused certain actions, but rather, in the sense that certain conscious states were, themselves, actions (i.e., mental acts).

### Conscious thought as an efficient cause of action

As experimental psychology moved away from consciousness and turned toward behavior in the early 1900s, explanations of “what we do” came to be couched in terms of efficient cause relationships between “stimuli” and “behavior.” And as cognitive psychology later challenged behaviorism's unwillingness to appeal to internal process (Tolman, 1951; Chomsky, 1959) it nonetheless adopted behaviorism's commitment to discovering efficient cause relationships. And now, instead of efficient cause residing between stimuli and responses, or vice versa, it has come to permeate the entire servo-mechanistic architecture that ultimately connects perceptual inputs to internal representations (i.e., cognitive structures) to behavioral outputs^1^.

Within such a servo-mechanistic framework, the relationship between consciousness and action control tends to be described such that conscious thoughts are modeled as causing actions. Another way to say this is that thoughts share an efficient cause relationship with actions. Despite the apparent obviousness of this claim, findings have come to the fore over the past few decades that severely challenge this idea. Wegner (2002) organizes these finding around Michotte's (1963) work on the perception of causality. Specifically, inspired by Hume (1739/1888) and his assertion that our sense of conscious agency constitutes yet another example of “perceived” causality based on contingent correlation (vs. metaphysical causality), Michotte discovered that our sense of causality was dependent upon the principles of priority (i.e., event A must precede event B for A to be experienced as the cause of B), consistency (i.e., the more event A is consistent with event B, the more event A is experienced as being the cause of event B), and exclusivity (i.e., the fewer event As there are, the more an event A is experienced as the cause of event B).

In Wegner's (2002) work, these principles translate into the idea that we perceive ourselves (i.e., our thoughts) to be the cause of our own actions to the extent our thoughts precede our actions (i.e., priority), our actions are consistent with our preceding thoughts (i.e., consistency), and our thoughts are the only available cause of our actions (i.e., exclusivity). Wegner then reports multiple examples of how violations of these principles lead to illusions of conscious will (i.e., feeling as though we caused actions we did not cause, or feeling as though we did not cause actions that we, in fact, did).

As regards priority, Wegner points to Kornhuber and Deecke's (1965) classic work on the Bereitschaftspotential (readiness potential), a negativity in the supplementary motor cortex that begins roughly 1 s prior to the initiation of a voluntary finger flexion. In Libet's (1985) classic work, he found that while the Bereitschaftspotential begins roughly 1 s *before* a movement, one becomes consciously aware of having planned a movement roughly 200 ms before the movement. This discrepancy is often interpreted as implying that the brain knows what one is planning to do before one is even aware of it. Wegner argues this constitutes a violation of the priority principle. That is, if thoughts cause actions, the “thought” of planning the tap should precede the onset of preparatory brain dynamics.

As regards consistency, Wegner (2002) cites Langer and Roth's (1975) finding that people are more likely to feel as though they controlled a chance event (e.g., they willed a particular number to result from the roll of a die) if they have previous experience successfully predicting such events. Wegner claims this to be evidence for the illusory nature of conscious will because people felt themselves to be in control of an event they were not in control of, simply because the final event was consistent with their preceding thoughts.

Finally, as regards exclusivity, Wegner reports research of his own (Wegner et al., 2003) in which participants completed a simple yes/no reaction time task while a confederate sat behind them. The confederate reached around the participant's torso and held her index fingers just above the fingers the participant was using to indicate yes/no responses. The confederates never made contact with the participant's fingers. And although the participants were accurate on 87% of the trials, they attributed 37% of the influence for the answers to the confederate. In short, the simple availability of the confederate as a potential cause of the response led the participants to experience a reduction in their own efficacy.

Wegner (2002) uses the above-mentioned experiments, as well as many, many others, to support the claim that conscious will is an illusion. That is, since these data so clearly reveal that our sense of agency (i.e., the feeling that our thoughts are the cause of our actions) is vulnerable to Michotte's (1963) principles of perceived causality, it must be the case that we are incorrect, and our sense of agency is actually an illusion. The true causes of our actions are unconscious, automatic associations between perception and action, what Bargh and Chartrand (1999) refer to as the “perception-action” link.

To be sure, there are contemporary cognitive scientists who disagree with the idea that conscious will is an illusion (see Baumeister et al., 2010). The point of addressing this issue so thoroughly at present is to propose that perhaps the reason conscious will is seen as being illusory is because certain researchers have committed themselves to an efficient cause approach to psychological functionality (i.e., how do we do what we do) that has historically led to the implicit assumption that thoughts cause actions. That is, it may be the case that thoughts did not evolve to cause actions, and the notion that conscious will is an illusion is a misconception one derives from a commitment to an illusory, efficient-cause architecture regarding the relationship between consciousness and action.

### Thought, action, and event control

Every year, millions of people all over the world watch professional soccer matches. During such matches, referees make judgments about the intentional states of players whenever the ball makes contact with a player's hand. The judgment has to do with whether or not the player intended (i.e., pre-specified) that the hand should hit the ball. While the anecdote might seem out of place, it nicely illustrates what is at stake in the conversation regarding the nature of conscious will. For if the referee decides the player acted intentionally, what is it that the player intended? Did the player pre-specify a particular movement of the hand or a particular outcome (i.e., hit the ball)?

William James (1890) believed that voluntary action had more to do with outcomes than limb movements:

I trust that I have now made clear what that ‘idea of a movement’ is which must precede it in order that it be voluntary. It is not the thought of the innervation which the movement requires. It is the anticipation of the movement's *sensible effects, resident, or remote, and sometimes very remote indeed*. (Volume 2, p. 521)

James' assertion that voluntary action involves the pre-specification (i.e., anticipation) of a movement's “sensible effects” is consistent with the notion that what is pre-specified during intentional action is the outcome, not limb movement. James then describes different levels of sensible effects: resident, remote, and very remote. “Resident” refers to the proximal, somatosensory, kinesthetic effects of movement. “Remote” refers to the distal effects of movement (e.g., seeing and feeling oneself make contact with a soccer ball). “Very remote indeed,” refers to effects beyond ones current context that one can pre-specify and work toward (e.g., going to the store to buy a bottle of milk, saving money to buy a new stereo, or becoming a college graduate).

Common to all three of these levels of sensible effects is the fact that (1) they can be pre-specified and therefore constitute intentionality, and (2) the pre-specification is of “effects” that will, at some point in time (i.e., proximal, distal, and abstract) result from movement. In short, inspired by James, it is my contention that “what we do” is best described as the pre specification and control of effects at multiple times scales, simultaneously; what I refer to as multi-scale effect control (MSEC). For example, as one dances a Tango with another, one simultaneously controls limb movements (i.e., proximal effects), one's distance from the partner (i.e., distal effects), and the larger-scale pattern of successfully completing an entire, pre-specified dance (i.e., abstract effects). All three levels are pre-specified and controlled continuously and simultaneously.

On the one hand, the notion that we pre-specify and control effects at multiple time-scales simultaneously seems at odds with the feeling that conscious will tends to involve one pre-specification at a time (e.g., pick up the pen, answer the question, walk to the store). In what follows, I review recent findings that reveal the brain continuously feeds memories of the past into the present as anticipation about the future, at multiple time scales simultaneously. In short, the anticipation of effects, resident, remote, and very remote indeed, constitutes a design principle of the brain.

### Multi-scale effect control and the brain

Over the past three decades, neuroscientists have discovered recursive connections between the cortex and the cerebellum that continuously render cortical activity anticipatory. Neurons in motor cortex, for example, project to neurons in the spinal cord as well to neurons in the cerebellum. These same cerebellar neurons receive inputs form the sensory neurons located in the limbs that are made to move by the associated motor neurons (Kawato et al., 1987). These cerebellar neurons project back to cortex. Thus, as one learns a particular limb movement (e.g., an infant learning to grasp a ball), and successful movements are repeated, successful *command-feedback regularities* become stored in these cortical-cerebellar networks such that when the infant later initiates such a movement, the cerebellar neurons are able to prime the motor cortical neurons before sensory feedback arrives from the moving limb. This is because the cortical-cerebellar networks have a time-cycle of 10–20 ms, while actual sensory feedback has a time-cycle of 120 ms. This faster-than-feedback time-cycle allows us to generate very fast, controlled body movements.

Kawato et al. (1987) refer to this cerebellar priming of cortex as *anticipatory motor error*, Clark (2001) and Grush (2004) refer to it as *virtual feedback*, and Paulin (1993) refers to it as *dynamic state estimation*. Quite often, these cerebro-cerebellar networks are referred to as *forward models* (Miall, 2003; Wolpert et al., 2003; Ito, 2005, 2008; Shadmehr and Krakauer, 2008; Golfinopoulos et al., 2009; Koziol and Lutz, 2013), and/or cerebellar control models (Koziol et al., 2011). Common to all these nomenclatures is the assertion that cerebellar-cortical networks are anticipatory and that the anticipation they entail derives from previous experience.

In addition to sharing recursive innervation with the motor cortex, the cerebellum also shares such connectivity with the reticular, autonomic, and limbic systems, as well as the prefrontal cortex, multimodal regions of the posterior parietal lobes, and the temporal lobes (Schmahmann, 2001). These recursive cerebro-cerebellar connections entail a two-step feedforward projection from cortex to the pons to the cerebellum, and a two-step feedback projection from cerebellum to thalamus to cortex (Schmahmann, 2001). Koziol et al. (2011) assert that the entire cortex is innervated by the cerebellum, save for the inferior temporal cortex, while Buckner et al. (2011) hypothesize the entire cortex is represented in the cerebellum, save very early vision and audition centers. Regardless of these small differences, it is clear the vast majority of the cortex shares recursive innervation with the cerebellum. Given these cortical projections to cerebellum are functionally segregated, it seems the brain entails a host of cerebellar control models (Koziol et al., 2011).

The discovery of memory-primed, prospective cerebro-cerebellar networks holds major implications for consciousness and action-control specifically, and cognitive science more generally. To begin, the existence of such networks constitutes evidence for James' (1890) assertion that what makes an action voluntary is the pre-specification of its “sensible effects.” While this idea conjures up images of an individual expending large amounts of conscious effort to imagine (i.e., pre-specify) what an action's sensible effects should be like, the notion of prospective cerebro-cerebellar networks illustrates how such pre-specifications are continuously fed to the cortex via its connections with the cerebellum. Activity in the cortex is continuously rendered prospective (i.e., anticipatory) as past experiences stored in cerebro-cerebellar networks are fed forward in the present as anticipations about what should happen next.

The discovery of prospective cerebro-cerebellar networks also provides support for James' (1890) assertion that “sensory effects” can be pre-specified at many different scales: resident (proximal), remote (distal), and very-remote, indeed (abstract). This implies that as we think, perceive, and act, cortical areas involved in such activities are continuously primed by past thoughts, past perceptions, and past actions stored in cerebro-cerebellar circuits. In short, cognition, perception, and action are all prospective, and what is “pre-specified” in each is the potential, eventual occurrence of an effect at an abstract, distal, or proximal time scale. Ito (1993) recognized this aspect of brain design decades ago and used it to argue that the neurodynamics underlying thought were of the same kind as those underlying movement control. The notion of a neurodynamic homology underlying thought and action is shared by many researchers (Schmahmann, 2001; Koziol et al., 2011; Ito, 2012; Koziol and Lutz, 2013), and it led Kinsbourne and Jordan (2009) to claim that anticipation constitutes a design principle of the brain.

Another point to make about such neurodynamic homology is the fact that all of these different event control systems function simultaneously. This means that future outcomes are being specified continuously at the proximal, distal, and abstract scale. For example, as one walks down a flight of stairs while talking to a friend and consciously anticipates what the friend will say next, one is simultaneously unaware of the fact that future outcomes are being generated for the feet; that is, until one steps out onto the floor and begins to fall forward because the floor is not there. During this moment of surprise, one is aware of the discrepancy between the pressure one was supposed to feel when the foot landed on the floor, and the unanticipated lack of pressure experienced because the floor was not there. It is in this moment of conscious error detection that one realizes she was unconsciously anticipating a certain amount of pressure on the bottom of the foot at a particular moment in the foot's trajectory. This unconsciously anticipated pressure on the foot is a pre-specified proximal outcome. It was generated as the cerebellum continuously and unconsciously primed the cortex with past patterns associated with negotiating stairs. In addition, the brain simultaneously generated conscious predictions about the conversation. In short, cerebro-cerebellar loops result in the cortex being continuously primed for events at multiple time-scales (i.e., proximal, distal, and abstract), simultaneously.

### Perception and action as multi-scale effect control

While on the one hand it seems appropriate to conceptualize motor control (i.e., proximal effect control) as being mediated via cerebro-cerebellar control loops, it is more challenging to conceptualize perception as being controlled via such loops. This is because traditional approaches to how we do what we do implicitly, if not explicitly, conceptualize perception as an attention-attenuated input that, in the end, is used to guide action. In what follows, I review research in the area of spatial perception in the hope of demonstrating how one might conceptualize perception as distal effect control.

Research on spatial perception clearly indicates we perceive the location of distal stimuli prospectively, in relation to the “sensible effects” we are pre-specifying for the distal stimulus. For example, it has been known for some time that the perceived vanishing point of a moving stimulus is localized beyond the actual vanishing point in the direction of stimulus motion (Hubbard, 1995, 2005). In addition, the magnitude of the spatial displacement (sd) varies with the laws of physics, in that the faster the stimulus movements, the larger the SD.

While SD is often accounted for in terms of representational momentum—the idea that evolution has endowed the brain with the ability to present dynamic as well as static properties—Jordan (2009) argues SD has more to do with planning dynamics than representational dynamics. In Kerzel et al. (2001) SD was eliminated, and in Jordan et al. (2002), SD actually became negative (i.e., participants perceived the stimulus to vanish behind its actual vanishing point) if participants were asked to fixate on a centrally located fixation cross as the stimulus moved across the screen, or moved around the fixation cross, respectively. That is, once participants were not allowed to track the movements of the stimulus with their eyes, which requires planning, forward SD vanished.

Further experiments reveal that the “planning” that gives rise to SD has to do with the movements of the distal stimulus (i.e., remote sensory effects according to James, 1890), not the movements of the body (i.e., proximal effects). For example, Jordan et al. (2002) asked participants to fixate on a centrally located fixation cross as a stimulus moved on a circular trajectory around the fixation cross. Half of the participants were asked to press a button as soon as the stimulus began to move (i.e., the cue condition). The other half was asked to press the button in order to make the stimulus vanish (i.e., the intention condition). This manipulation resulted in two groups of participants who were generating the same proximal effects (i.e., fixate on a fixation cross and press a button) in order to obtain different distal effects (i.e., respond to the stimulus' onset or make it vanish). In the cue condition the pre-specified distal effect referred to the initial position of the stimulus, while in the intention condition, it referred to final position.

When participants pressed the button the stimulus vanished. Participants then indicated the perceived vanishing point. Analyses revealed that those responding to the onset of the stimulus (i.e., the cue condition) saw it vanish behind the actual vanishing point, in the direction of the initial position, while those who pressed the button to make the stimulus vanish (i.e., the intention condition) saw it vanish precisely where it had vanished. This difference in perceived vanishing points is consistent with the assertion that the influence of planning on spatial perception derives from the distal effect (i.e., stimulus movements) the participant is planning, not the body movements (i.e., proximal effects) generated in order produce the distal effect. Both groups were specifying and controlling the same proximal effects (i.e., hold the eyes in a certain position and move the finger in a certain way), but they were doing so for different distal reasons. For those in the cue condition, the specified distal effect (i.e., press the button as soon as the stimulus appears) referred to the initial position of the distal stimulus, and the perceived vanishing points were attracted backward toward this initial position. For those in the intention condition, the specified distal effect (i.e., press the button in order to make the stimulus vanish) referred to the final position of the distal stimulus, and given the vanishing point was known by the participants because they pre-specified it and produced it, there was no SD.

Collectively, the data of Kerzel et al. (2001) and Jordan et al. (2002) indicate that the an important portion of the SD experienced in studies involving oculomotor tracking (Hubbard, 2005) derives from the planning required to keep the eyes aligned with the movements of the stimulus. This is consistent with James' (1890) assertion that we are able to pre-specify remote (i.e., distal) effects. Another important aspect of these distal pre-specifications is that they have to be generated continuously if one it to successfully track the moving stimulus. To be sure, no one experiences himself or herself as consciously willing the eyes to move. Rather, what one experiences is the pre-specification and sustainment of “track the stimulus.” How it is that one actually accomplishes the tracking is simply outside of one's conscious awareness. This is interesting, for it implies that the anticipated stimulus position (i.e., the specified distal effect) is not being generated consciously. This implies that while one is consciously pre-specifying “track the stimulus” the anticipated stimulus positions are being generated outside conscious awareness.

In order to better understand how it is one can learn to unconsciously pre-specify distal effects, Jordan and Hunsinger (2008) conducted an experiment in which one participant controlled stimulus movements back and forth across a computer screen via right and left button presses on a keyboard while another participant, who could neither see nor hear the controller, observed the movements of the stimulus on a separate monitor. At some point during the trial the stimulus unexpectedly vanished and the observer moved a crosshair to the location on the monitor where she saw the dot vanish. After forty trials (i.e., Phase 1), the two participants switched roles and the participant who previously controlled the stimulus now indicated perceived vanishing points (i.e., Phase 2). Analysis revealed that participants with previous control experience produced significantly larger SD than participants having no such experience.

In another experiment, Jordan and Hunsinger (2008) investigated the aspects of Phase 1 control experience that led to later changes in perception. Specifically, they replicated Experiment 1 except for the fact that during Phase 1, an observational learner sat next to the controller. In Phase 2, the observational leaner switched places with the Phase 1 naïve observer (i.e., the Phase 1 naïve observer controlled the stimulus while the Phase 1 observational learner observed stimulus movements and indicated perceived vanishing points). In addition, during Phase 1, half of the observational learners were allowed to see the movements of the stimulus on the computer screen as well as hear and see the button presses the controller made while controlling the stimulus movements. In contrast to these “full access” participants, the other half of the observational learners were denied access to the actions of the controller (i.e., a board prevented them from seeing the key presses while headphones prevented them from hearing the key presses). Analyses revealed that the Phase 2 SD from the “full access” observational learners was larger than that of the “no action access” observational learners. In addition, the SD values of the two groups basically replicated the SD pattern of Experiment 1, with “no action access” participants producing SD similar to that of naïve observers, and “full access” participants producing SD similar to that of observers having previous control experience. In short, observational learners who were given access to the proximal and distal effects generated by the controller (i.e., key presses and stimulus movements, respectively) later perceived the stimulus movements in the same way it was perceived by those who had actually, previously controlled it.

Jordan and Hunsinger (2008) accounted for these finding by asserting that during Phase 2 observation, the moving stimulus activated the distal effect planning (i.e., planning of stimulus movements) the participant had learned to generate while controlling the stimulus during Phase 1. That is, having learned to control the distal event in Phase 1 (i.e., the movements of the stimulus), perception of the stimulus in Phase 2 activated Phase 1 control memories such that the participant experienced the stimulus in terms of the control dynamics learned during Phase 1.

Recent findings in cognitive neuroscience shed light on how “remembered control dynamics” can be activated during perception. First, areas of the cortex involved in *planning* distal events (i.e., pre-motor cortex) are also involved in *detecting* distal events (Rizzolatti et al., 1996; Hommel et al., 2001). This reveals that we perceive distal events in terms of the plans we would generate to produce the distal event ourselves. Second, a finding previously mentioned in the present paper, the vast majority of the cortex shares recursive innervation with the cerebellum (Miall, 2003; Koziol et al., 2011). Collectively, these finding indicate that (1) seeing is planning, and (2) planning activated by distal events is continuously, prospectively primed by recursive cerebro-cerebellar memories. Thus, while participants controlled the stimulus during Phase 1, patterns in the planning states they generated for the stimulus (e.g., make it accelerate, make it coast across the screen, make it decelerate, make it stop and change direction) altered the cerebro-cerebellar dynamics generating these plans such that during later observation, the movements of the stimulus gave rise to pre-motor planning dynamics that were continuously, prospectively primed by remembered planning patterns embedded in cerebro-cerebellar dynamics. To be sure, during later observation participants did not have to consciously generate anticipated stimulus locations. Rather, they consciously activated “watch the stimulus,” and given that distal-event detection (i.e., perception) and distal-event planning (i.e., distal effect planning) share neural overlap, they “perceived” the stimulus movements in terms of distal-event plans that were continuously primed by cerebro-cerebellar memories. Thus, while “watch the stimulus” was consciously pre-specified, conscious anticipation of stimulus positions was unnecessary, for they were provided by cerebro-cerebellar memories.

While the notions that (1) distal-event planning and detection share neural overlap, and (2) continuous priming of planning via cerebro-cerebellar memories, collectively provide an account of why observers with previous control experience give rise to larger SD than naïve observers, they do not explain the differences in SD between the “full access” and “no action information” observational learners. As an account, Jordan and Hunsinger (2008) propose that full access observational learners developed planning memories like those of controllers because while they watched the controller control the stimulus during Phase 1, they had continuous access to both the movements of the stimulus (i.e., the distal effect) and the key presses the controller made in order to control the movements (i.e., the proximal effect). This assertion is supported by data indicating that in addition to pre-motor cortical areas being involved in both planning and detecting distal events (i.e., what is often referred to *mirroring*), there are parietal cortical areas (i.e., PF) that are involved in both the planning and detection of proximal events (i.e., body movements). Iacoboni (2005) proposes that the pre-motor mirroring systems and the parietal mirroring systems, along with STS located in the temporal lobe, collectively constitute a mirroring system that affords us the ability to imitate and understand the actions of others. Iacoboni further proposes that while the planning which occurs in pre-motor cortex refers to distal events (e.g., grasp a raisin), the planning in parietal cortex has more to do with proximal effects (i.e., the anticipated somatosensory feedback of moving an effector). This assertion is based on findings that indicate that as one simply observes meaningful and meaningless actions, frontal mirroring activation is more prominent in the former, while parietal activation is more prominent in the latter (Grezes et al., 1999). This is because the parietal system is involved in the analysis of body movement. This “frontal-parietal” division of labor is further supported by the finding that that both systems are active during the observation of meaningful and meaningless actions if one has the goal of imitating the observed action.

Jordan and Hunsinger (2008) utilized Iacoboni's frontal-parietal-STS mirroring system theory of imitation as an account of why observational learners with access to the actions later produced large SD similar to that of those having previous control experience. Specifically, they assert that while observing the controller control the movements of the stimulus via button presses, the movements of the stimulus activated the frontal mirroring system (i.e., distal-effect system—Jordan, 2003) while the sight and sound of the finger movements activated the parietal mirroring system. These frontal-parietal activations would have activated their associated cerebellar recursions. Given these mirroring systems are involved in both pre-specification (i.e., planning) and detection (i.e., perception), their continuous activation via observation could have driven the observer through the same planning states the controller was undergoing. It's as if the proximal-distal pattern generated by the controller hijacked the multi-scale planning states of the observer and, in a sense, drove the observer's proximal-distal cerebro-cerebellar systems as if the observer were generating the planning states endogenously. It seems possible that the repeated, exogenous activation of these systems led to changes in the observer's cerebro-cerebellar systems such that during later observation, the movements of the stimulus drove the observer's planning states as if they had actually had previous control experience. Observational learners who did not have access to the controller's actions, would not have been able to experience the proximal-distal patterns generated by the controller (i.e., they only had access to the distal pattern—the movements of the stimulus). Hence, they did not have the opportunity to learn a proximal-distal model, and during later observation, simply experienced the stimulus much like a naïve observer.

Collectively, the work of Kerzel et al. (2001), Jordan et al. (2002), and Jordan and Hunsinger (2008) lead to two very important implications regarding multi-scale event control: (1) multiple effects at different scales (i.e., proximal and distal) are pre-specified continuously and simultaneously, (2) the pre-specified effects are generated unconsciously via remembered “planning” dynamics embodied in cerebro-cerebellar loops. In addition, given that cortical areas involved in detecting events are also involved in pre-specifying distal events, it seems difficult to sustain the traditional practice of conceptualizing perception as an attention attenuated input that is used to guide action. The finding that mirroring systems are involved in both the detection and pre-specification of distal effects indicates these systems simultaneously contain both the pre-specified distal effect (i.e., the goal) and the current state of the distal event (i.e., feedback). Thus, it seems evolution has left us with a rather elegant solution to controlling distal effects. Instead of developing one system for *doing* and another for *seeing*, as is assumed in traditional approaches of psychological functionality, evolution has endowed us with a host of systems that are able to pre-specify and detect distal events simultaneously.

### Cognition as multi-scale event control

In addition to proximal and distal effects, however, participants in the above-mentioned studies were simultaneously pre-specifying and generating effects that could be labeled as cognitive. For example, all of the participants were pre-specifying and sustaining the abstract effect of complying with the experimenter's instructions. This is an abstract effect, what James (1890) referred to “very remote indeed” because it is a pre-specification of the how the participant will configure herself in the current context.

There are infinite degrees of freedom in terms of the proximal-distal pattern of effects one can pre-specify and sustain in a given context. The participant could have hopped on one leg across the room, or curled up into a corner and read a book. Both of these proximal-distal configurations were afforded by the laboratory context. Participants were able to inhibit all the other proximal-distal options the context afforded and, instead, produce the “make the stimulus move across the screen by pressing buttons” option requested by the experimenter, because they were able to pre-specify it (i.e., they constrained their proximal and distal effect systems toward controlling the movements of the stimulus) and sustain it (i.e., they prevented their proximal and distal effects systems from producing a different configuration). As any caregiver knows, getting a child to organize himself in a particular way in a particular context (e.g., clean up his room) is very difficult. Fair et al. (2007) report that the cinguloopercular system believed to underlie set maintenance (i.e., the ability to focus on a specific task—maintain a specific abstract effect—for an extended period of time) segregates itself developmentally from the frontoparietal system believed to underlie adaptive online task control (i.e., proximal and distal effect control). Thus, by the time a college student participates in an experiment, she has already developed neural systems that afford abstract effect control.

To be sure, what a student is “doing” in an experiment is even more abstract (i.e., remote) than complying with instructions. For as students comply with instructions, they are actually doing so in order to sustain an even more abstract (i.e., more remote) effect; namely, receiving extra credit or monetary payment for participating in an experiment. And what is more, they are pre-specifying and sustaining the “extra credit” abstract effect in order to work toward achieving the even more abstract effect (i.e., very remote indeed) of receiving a particular grade in a course.

The point being made here is that when a person participates in an experiment, they are doing significantly more than theories of action, perception, and/or cognition often give them credit for doing. Specifically, while participants in the previously mentioned studies were pre-specifying and producing the distal effect regarding the distal stimulus (i.e., make the stimulus move back and forth across the screen), they were *simultaneously* pre-specifying and generating multiple proximal effects (e.g., move your eyes in a manner that allows you to track the stimulus, and move your fingers in ways that result in the buttons being pressed). To be sure, they were actually pre-specifying and generating many, many other proximal effects all at the same time, such as hold the body in a particular position, keep the hand configured in a way that affords fast button presses, and keep the head positioned toward the computer monitor.

One could try to make the case participants were “perceiving,” “acting,” and “thinking.” But in reality, as the above-stated examples indicate, they were doing so much more (i.e., pre-specifying and sustaining a constellation of multi-scale effects), and they were doing all of it at the same time.

## How we do it

Given that persons pre-specify and sustain multiple effects at multiple time-scales simultaneously, it is not clear to what extent the concepts “action,” “perception,” and “cognition” are terribly useful in a scientific context. Traditional assumptions that frame perception as input, action as output, and cognition as intermediary processing, fail to acknowledge the cerebro-cerebellar homology that underlies the various levels of effect control. This leads them to overlook the fact that all levels of effect control entail pre-specification (i.e., planning) and detection (i.e., perception).

### Planning and control in msec

To be sure, “planning” looks different in this framework in that it (i.e., planning) takes place continuously at multiple levels of effect control as the cerebellum prospectively and continuously primes the cortex. “Control” also looks different within the framework of MSEC because it (i.e., control) does not mean “cause” in the efficient cause sense that one level of effect control (e.g., conscious thought) “causes” changes in another (i.e., action) in the same way one billiard ball “causes” another to move (Jordan and Ghin, 2007). Rather, within the framework of MSEC, different levels of effect control *constrain* each other. That is, the more proximal scales of effect control (e.g., moving one's hand a particular way, or positioning one's body in a particular configuration) find themselves prospectively and continuously *constrained* toward the generation of pre-specified distal effects (e.g., press the buttons or sit in front of the computer, respectively). And these distal-effect systems (Jordan, 2003; Clark, 2007) find themselves prospectively and continuously constrained by more remote, abstract effect systems (e.g., comply with the experimenter's instructions or obtain extra credit points for a course) as well as proximal effect control systems.

*Constraint* in this sense means that the cortical areas involved in different levels of effect control influence each other continuously via neural recursion. For example, Fair et al. (2007) report that during the developmental segregation of the cinguloopercular system (i.e., the system believed to underlie set maintenance) and the frontoparietal system (i.e., the system believed to underlie online task control), short-range neural connections between closely adjacent brain regions within each system “grow down” (i.e., decrease) with age, while long-range functional connections between the systems “grow up” (i.e., increase). In addition, they speculate,

These developmental dynamics may represent a learning mechanism whereby precursors to adult task sets are originally derived from more available signals generated by regions of the more rapidly adaptive control network (i.e., frontoparietal). In this sense, the performance of tasks with novel components would rely more heavily on rapidly adaptive control generated by the frontoparietal network. With greater age, and therefore greater experience, stored task sets may be retrieved and stably maintained throughout the task epoch by the cinguloopercular network. (p. 13511)

This developmental increase in long-range neural connectivity allows different levels of effect control to constrain, not cause, each other because at any given moment, the activity of a given neural area is modulated continuously by both long-range and short-range projections. Thus, the activity configuration in a given cortical area at any given time constitutes an emergent, dynamic compromise among all the forces impinging upon the neurons that constitute that cortical area. In short, the extreme level of recursion in brain organization makes if difficult, if not impossible, to make coherent “efficient-cause” assertions regarding brain dynamics in general, let alone the type of influence one level of event control shares with another, specifically.

MSEC's proposal to conceptualize brain dynamics in terms of constraint as opposed to efficient cause is consistent with Rosen's (1991) assertion that the dynamics of biological systems in general are simply closed to efficient cause. It is also consistent with Van Orden and Holden (2002) assertion there does not exist a causally isolated level of brain dynamics capable of mediating efficient cause relationships between isolated content vehicles. Rather, brain dynamics are inherently “interaction-dominant” in that activity in all neurons, as well as neural areas, is continuously modulated by the activity taking place in a plethora of other neurons and neural populations. To continue the recursion, recursive brain dynamics are continuously modulated by body and world dynamics, just as body and world dynamics are continuously, recursively, modulated by brain dynamics.

### Multi-scale recursion, action control, and consciousness

In the midst of all this multi-scale recursion (i.e., constraint), it becomes increasingly difficult to sustain “efficient cause” approaches to “how we do it.” Neither psychological functions, neural networks, nor neurons are causally isolated. As a result, assertions regarding whether or not there exist efficient cause relationships between thought and action might simply be outdated. And experiments that reveal persons to be capable of feeling as though they caused events they did not, or as though they did not cause events they actually did, might be misinterpreted.

Instead of such data revealing a delusion of control (Wegner, 2002), they might reveal intervals of uncertainty that emerge spontaneously as one controls multiple effects simultaneously. For example, Knöblich and Kircher (2004) asked participants to control the trajectory of a dot presented on a computer monitor. They did so by moving a stylus on a writing pad capable of detecting and codifying stylus movements. Participants could not see their hand movements. The specific task was twofold: first, they were to make the dot move in such a way that its movement through the 12:00 position was synchronized with the presentation of a temporally predictable tone. Second, they were to lift the stylus from the pad if, at any moment, they detected a difference between stylus and dot movement.

To assess the participants' sensitivity to perturbations of visuo-motor coordinations, the experimenters manipulated the relationship between the movement dynamics recorded on the digital pad and the visual effects displayed on the monitor such that on the fourth cycle of a given trial, the velocity of the dot was increased relative to the movements recorded on the pad. As a result, in order to execute a visual circle, participants had to basically draw an ellipse. Results indicated that participants did not become aware they were drawing ellipses versus circles until the velocity of the stimulus was increased by 50% of its initial value.

From the traditional perspective, one might claim that during the period in which participants were not aware of the discrepancy between the ellipses they were generating with their hands and the circles they were generating on the monitor, they were suffering a delusion of control. That is, one might assert participants were experiencing an illusion of conscious will because they were intending to produced circles with their hands but were actually producing ellipses. However, it might also be the case that the participants' proximal and distal control systems were functioning properly (i.e., the proximal systems were controlling hand movements—pre-specified and achieved kinesthetic feedback—while the trajectory of the hand movements was continuously constrained by distal effect systems). In this sense, one might propose that proximal and distal effect systems are coupled in such a way that the function of the latter is not to “cause” the former, but rather, to “constrain” the former toward a specific distal outcome—draw a circle.

Given that *constraint* takes time as the neurodynamics supporting one level of event control influence the neurodynamics of another, one should not be surprised to find temporal windows (i.e., psychophysical intervals of uncertainty) during which a distal-event system is “unaware” of the faster time-scale dynamics of the proximal-event systems the former is constraining. Dennett (1991) said much the same thing in his critique of Libet's (1985) paradigm. Specifically, he claimed that the temporal order, or sequence, in which the nervous system distributes information is not dictated by the order in which the information is transduced by the sense organs. Rather, it is dictated by the temporal constraints imposed by the on-going control of the body in space-time. Dennett refers to these constraints as “temporal control windows” and contends that the nature of these windows is a function of the relevant sensory-motor coordination.

When we are engaged in some act of manual dexterity, “fingertip time” should be the standard; when we are conducting an orchestra, “ear time” might capture the registration. (p. 162)

According to MSEC, different event control systems will have different “temporal control windows,” and results such as those obtained by Knöblich and Kircher (2004) emerge out of the multi-scale temporal control windows demanded by a certain task.

This idea of yoked, yet distinct systems that function simultaneously and mutually constrain one another is part and parcel to distinctions vision researchers frequently make when referring to Milner et al.'s (2006) vision for perception, versus vision for action distinction. The major difference between MSEC and Milner and Goodale's model is that, in the latter, “vision for action” and “vision for perception” basically reduce to “visual *input* for moving” and “visual *input* for seeing,” respectively. This notion that perception (i.e., seeing) constitutes *input* still permeates both contemporary philosophical and psychological discussions regarding the ventral-dorsal distinction (Clark, 2007; Milner et al., 2013). In MSEC, perception is not *seeing*; it is not *input*. Rather, it is the pre-specification and detection of distal events. In short, it is distal-effect control. And what is more, all levels of effect control entail pre-specification (i.e., planning) and detection (i.e., perception). So to refer to one brain area as a “doing” area and another as a “seeing” areas prevents one from recognizing that all such areas are “doing” something (i.e., controlling effects) via the same cerebro-cerebellar homology, just at different, yet yoked, time scales.

## Why it has meaning

To be sure, conceptualizing “how we do it” in terms of multi-scale systems sharing recursive interactions is not new. This idea is espoused by many theorists in both the dynamical systems camp (Clark, 1997, 2000; van Gelder, 1998; Juarrero, 1999; O'Regan and Nöe, 2001; Myin and O'Regan, 2002; Van Orden and Holden, 2002) and the computationalist camp (Powers, 1973; Kawato et al., 1987; Meyer and Kieras, 1997a,b). What distinguishes MSEC from these other approaches is the manner in which it conceptualizes the nature of the multi-scale dynamics. Specifically, MSEC is actually a sub-component of a larger theoretical framework known as Wild Systems Theory (WST). According to WST, living systems are comprised of multi-scale systems of self-sustaining work. “Self-sustaining work,” in this context refers to patterns of energy transformation that produce products that feedback into and sustain the work that produced the product in the first place. [For a thorough description of WST and its take on self-sustaining work please see Jordan and Ghin, 2006, 2007; Jordan, 2008; Jordan and Heidenreich, 2010; Jordan and Vinson, 2012]. According to Jordan and Vinson (2012):

At the chemical level, self-sustaining work has been referred to as autocatalysis (Kauffman, 1995), the idea being that a self-sustaining chemical system is one in which reactions produce either their own catalysts or catalysts for some other reaction in the system. At the biological level, self-sustaining work has been referred to as autopoiesis (Maturana and Varela, 1980), again, the idea being that a single cell constitutes a multi-scale system of work in which lower-scale chemical processes give rise to the larger biological whole of the cell which, in turn, provides a context in which the lower-scale work sustains itself and the whole it gives rise to (Jordan and Ghin, 2006). Hebb (1949) referred to the self-sustaining nature of neural networks as the “cell assembly,” the idea being that neurons that fire together wire together. Jordan and Heidenreich (2010) recently cast this idea in terms of self-sustaining work by examining data that indicate the generation of action potentials increases nuclear transcription processes in neurons which, in turn, fosters synapse formation. At the behavioural level, Skinner (1976) referred to the self-sustaining nature of behaviour as operant conditioning, the idea being that behaviours sustain themselves in one's behavioural repertoire as a function of the consequences they generate. Streeck and Jordan (2009) recently described communication as a dynamical self-sustaining system in which multi-scale events such as postural alignment, gesture, gaze, and speech produce outcomes that sustain an ongoing interaction. And finally, Odum (1988) and Vandervert (1995) used the notion of self-sustaining work to refer to ecologies in general. (p. 235)

WSTs assertion that organisms are constituted of multi-scale self-sustaining work reveals the dynamic homologies that transcend both the phyla and the nesting of multi-scale energy-transformation systems that constitute a single organism. From plants, to neurons, to behavior, to persons, to human societies, increasingly complex systems of work (i.e., energy transformation) have evolved precisely because the very work of which they are constituted, is self-sustaining. That is, the work produces catalysts for either the work itself, or some other level of work in the multi-scale system.

In addition to revealing the multi-scale homologies that constitute an organism, WST's notion of multi-scale self-sustaining work affords a conceptual reframe of the context in which organisms sustain themselves. In traditional accounts, nature is conceptualized as being physical, and phenomenal properties such as meaning, value, and consciousness are conceptualized as either identical with the physical (i.e., identity theory), emergent from the physical (i.e., emergentism), as an informational property of causal relations (i.e., functionalism), or as an aspect of reality other than the physical (i.e., double-aspect theory and property dualism). Again, as was stated at the outset of the present paper, in all of these positions, phenomenal properties do not constitute a logically necessary aspect of the causal story. As a result, phenomenal properties do not enter into a scientific, causal description of what we do and how we do it. In short, consciousness is an epiphenomenon.

Within WST however, “nature” is conceptualized as a self-organizing energy-transformation hierarchy (Odum, 1988; Vandervert, 1995) within which “the fuel source dictates the consumer” (Jordan and Ghin, 2006). What this means is that any system that sustains itself on a given fuel source (e.g., plants on sunlight, herbivores on plants, or carnivores on herbivores) must be constituted in such a way that it is capable of addressing all the constraints involved in capturing that fuel source. Given this necessary connection between a consumer, its fuel source, and the context in which the two exist, it seems appropriate to claim that an organism constitutes a multi-scale, self-sustaining embodiment of the constraints entailed in taking in, transforming, and dissipating its fuel source. Said another way, organisms are self-sustaining embodiments of the contexts in which they phylogenetically and ontogenetically emerged.

Conceptualizing organisms as embodiments of context is an important move for WST because it provides a means of conceptualizing organisms as inherently meaningful. Specifically, if an organism constitutes an embodiment of context, then it is naturally and necessarily “about” that context. That is, its internal dynamics are phylogenetically and ontogenetically emergent from the energy-transformation hierarchy in which it has sustained itself. As a result,

… there is no epistemic gap between an organism and its environment. Organisms do not need to be “informed” by environments in order to be about environments because they are necessarily “about” the contexts they embody. Rather, what self-sustaining systems need do is sustain relationships with the contexts in which they are embedded in ways that lead them to sustainment. According to WST, meaning is constitutive of embodied context (i.e., bodies). As a result, living systems are necessarily meaningful (Jordan, 2000a), not because a body is alive or dead, because it is physical, or because it is biological. Living is meaning because it is sustained, embodied context. (Jordan and Vinson, 2012, p. 9)

### Embodied context, action-control, and consciousness

Given the notion of “embodied context,” WST asserts that the phenomenon we refer to as consciousness is actually a phylogenetically scaled-up recursion on the embodied aboutness inherent in all organisms. What determines the distality of the aboutness (i.e., the level of conscious awareness) an organism entails varies with the distality of the contexts in which the organism can pre-specify outcomes and work to sustain those outcomes: resident, remote, and sometimes very remote indeed. As an example of species differences in the scale of event control, while my dog and I can jointly sustain the outcome of playing tug-of-war in the hear-and-now, my dog is not able to organize himself in the hear-and-now in order to play tug-of-war again at the same time tomorrow. Dogs are not able to pre-specify the very remote effect, “tomorrow,” and therefore, cannot sustain a relationship with it. From this perspective, I am able to pre-specify and sustain relationships with contexts that are vastly more “remote” than those of my dog.

On the one hand, it may seem that the obvious account of why different species sustain effects at different time-scales is a neural one; organisms capable of sustaining increasingly abstract effects (e.g., “tomorrow,” “next June,” or “forever”) can do so because they have more sophisticated brains. On the other hand, WST proposes it is more than just brains. Rather, consistent with Oyama (2000), Jordan (2008) asserts that the sustainment of abstract contexts necessitates the emergence and sustainment of external contexts such as language and technology specifically, and culture, in general (what Oyama refers to as developmental contexts). It is within this entire multi-scale, contextually emergent, self-sustaining system of work that nested sub-systems (i.e., individual humans) are able to generate and sustain abstract contexts. Again, consistent with Oyama, from this perspective, infants inherit much more than genes. In short, they inherit a culture.

Within such a multi-scale, self-sustaining transformation hierarchy, the issue of action-control and consciousness is about so much more than the issue of whether or not conscious thoughts cause actions. To be sure, consciousness (i.e., embodied aboutness, or embodied context) does exist, but not in the way it is thought to exist within traditional frameworks that conceptualize consciousness as being opposed to unconsciousness. Again, within WST, “aboutness” is a constituent property of all self-sustaining embodiments of context. All self-sustaining systems *are* abountess (Jordan, 2000a). (See Jordan and Vinson, 2012, for a thorough analysis of how these ideas are related to the non-living systems). Thus, according to WST, the issue of consciousness and action control as it is traditionally framed, is framed in WST in terms of the effects that are most prescient during any given movement of multi-scale effect control. For example, while conversing with a friend and walking down a flight of stairs, sensed foot pressure is not prescient (i.e., it is not within one's currently reportable conscious states) until there is an error (i.e., pre-specified and attained foot pressure do not match). As one starts to fall because the predicted floor is not there, sensed foot pressure becomes prescient; it comes to dominate immediate, reportable consciousness as one struggles to avoid falling down the stairs.

From this perspective, consciousness does not reside at a particular level of event control. Instead, it is fluid and makes its way transiently to different levels of effect control as different effects become contextually prescient (Jordan, 2003). This implies that consciousness has more to do with managing relationships across different levels of effect control. In a test of this idea, Kumar and Srinivasan (2013) asked participants to use a joystick to aim at targets on a computer screen. Target trajectory entailed one of three levels of random perturbation. After the participant pulled the trigger on the joystick, a stimulus appeared at the targeted location, and the participants indicated (1) how much time passed between the trigger pull and the appearance of the stimulus, and (2) how confident they were that they themselves were the author of the action. Results revealed that if participants missed the target (i.e., the more distal effect was not achieved), estimates of the action-stimulus interval were significantly correlated with the actual action-stimulus interval as well as the degree of noise in the target movements. Specifically, as the amount of noise in the target movements decreased, time estimates also decreased. This temporal attraction of the timing of a post-action stimulus toward the moment of the action is referred to as intentional binding (Jordan, 2000b; Haggard et al., 2002), and it is assumed to constitute an implicit measure of one's sense of agency. If, however, the participant hit the target, the pattern changed. Specifically, estimates of the action-stimulus interval were significantly correlated with the action-stimulus interval (i.e., intentional binding occurred), but they were not correlated with the degree of noise in the stimulus. This indicates that once the distal effect is achieved, one's consciousness is more about the achieved distal effect than the constraints that had to be addressed by the proximal control systems as they worked to achieve the distal effect.

The idea that consciousness ebbs and flows across different levels of effect control has much in common with Vallacher et al.'s (1989) action-identity theory, which assumes that there are many different ways to cognitively identify (i.e., represent) a given action, but only one identification tends to be prepotent for the actor at any given moment:

… although talking, for example, could be identified as sharing information, expressing an opinion, influencing someone, passing time, or choosing words, the actor is likely to have in mind only one of these identifications. (p. 199)

The notion of consciousness working as a manager across levels of effect control is also consistent with Baars' global workspace hypothesis (1988), which asserts that the purpose of consciousness is to make the contents regarding a specific conscious experience massively available to a host of unconscious brain processes so that these latter brain processes can be brought to bear on the immediate situation. From this perspective, consciousness ebbs and flows across different contents as different problems emerge for the system in real time. Morsella (2005) proposes a similar view in which the purpose of phenomenal (i.e., conscious) states is the resolution of conflicts between action plans as different action systems compete for expression through the skeletal muscular system, what he refers to as PRISM (i.e., parallel responses into skeletal muscle).

Common to PRISM, Global Workspace Theory, and WST is the idea that potential conflicts among competing actions (i.e., effect control systems) need to be sorted out by the system. From the traditional perspective, this might be taken to mean that a certain conscious state intervenes and causes a particular action to be expressed. From the perspective of WST, it means that at any given moment, the pattern of multi-scale effects one works to control emerges spontaneously and continuously out of both exogenous influences that activate pre-specifications of past effect-control episodes via cerebro-cerebellar systems, and the endogenous constellation of constraint that builds up over the life course across different levels of effect control. Imamizu and Kawato (2009) review a host of empirical findings that are consistent with the idea that moment-to-moment changes in effect-control dynamics, what they refer to as the switching of internal models, is brought about my the continuous, exogenous and endogenous modulation of internal models (i.e., cerebellar control models).

On the one hand, GWT and PRISM seem to have the advantage of Occam's razor. They provide a clear, causal story of how changes in a physical system like the brain are associated with conscious states. On the other, WST overcomes the potential epiphenomenalism inherent in the physicalism of both GWT and PRISM, because WST provides an account of what consciousness is and why it is necessary. However, according to WST, consciousness is not necessary because it helps physical brains sort out potential actions. Rather, it is necessary because it is what organisms are.

## Conclusions

The purpose of the present paper was to present an approach to the issue of *consciousness* and *action control* that, in the end, challenges the utility of concepts such as *consciousness* and *action control* in a science of what we do and how we do it. Traditional models assert we do things such as act, perceive, think, attend, and remember. While these concepts have great utility in daily life, from which they emerged, it is my contention they are not complex enough to address the host of hypercomplex regularities cognitive science has discovered over the past 30 years. Brains specifically, and living systems in general, have turned out to be closed to efficient cause (Rosen, 1991) and interaction-dominant (Van Orden and Holden, 2002). Action oriented areas of the brain have turned out to be simultaneously perceptual (Miall, 2003), and moment-to-moment experience finds itself having a prospective, anticipatory edge as memories of the past continuously prime those areas of the cortex we once thought served the purpose of informing us about the present. What we do and how we do it turns out to be continuous, multi-scale, and wild. By wild I do not mean out of control. To the contrary, I mean massively in control. Not like a closed system such as a robotic arm placing hyper accurate welds on an assembly line, or a computer code parsing chunks into appropriate sectors. Rather, like an open system such as a bird in flight, whose wing dynamics absorb and resist the multi-scale wind patterns it encounters in real time, not because it has to control its flight, but because controlling flight is what it is.

### Conflict of interest statement

The author declares that the research was conducted in the absence of any commercial or financial relationships that could be construed as a potential conflict of interest.

## Footnotes

^1^To be sure, there have been those who have critiqued experimental psychology's reliance on efficient cause explanations all throughout its history. These critiques have come primarily from researchers espousing a more dynamic approach to psychological functionality including the Gestalt psychologists (Ash, 1995), the New Realists such as Holt and Gibson (Charles, 2011), and a host of contemporary researchers making use of dynamical systems theory such as van Gelder (1998); Van Orden and Holden (2002), and Coey et al. (2012). These will be discussed in the section below entitled, “How we do it.”
